# Deciphering *Trypanosoma lainsoni* kDNA minicircles: insights into genetic diversity, mRNA editing, and molecular diagnosis

**DOI:** 10.1051/parasite/2026034

**Published:** 2026-06-03

**Authors:** Juan José Aguirre, Noelia Floridia-Yapur, Fanny Rusman, Soledad Hodi, Tatiana Ponce, Anahí Guadalupe Diaz, Gonzalo Greif, Luisa Berná, Carlos Robello, Patricio Diosque, Nicolás Tomasini

**Affiliations:** 1 Unidad de Epidemiología Molecular (UEM), Instituto de Patología Experimental “Dr. Miguel Ángel Basombrío”, Universidad Nacional de Salta-CONICET Salta Argentina; 2 Laboratorio de Interacciones Hospedero-Patógeno – UBM, Institut Pasteur de Montevideo Montevideo Uruguay; 3 Unidad de Bioinformática, Institut Pasteur de Montevideo Montevideo Uruguay; 4 Laboratorio de Genómica Evolutiva, Facultad de Ciencias, Universidad de la República Uruguay; 5 Departamento de Bioquímica, Facultad de Medicina, Universidad de la República Montevideo Uruguay

**Keywords:** *Trypanosoma lainsoni*, kDNA, Minicircles, Next Generation Sequencing

## Abstract

The mitochondrial genome of kinetoplastids, organized in a unique DNA net called the kinetoplast, presents significant complexity compared to other eukaryotic mitochondrial genomes. Minicircles, which constitute over 90% of the kinetoplast mass, are essential for the post-transcriptional editing of maxicircle transcripts. This study focuses on *Trypanosoma lainsoni*, a trypanosomatid first discovered in northern Brazil and later reported in Argentina. Utilizing a combined second and third generation sequencing approach, we conducted an in-depth analysis of the kDNA minicircles of three Argentinian isolates of *T. lainsoni.* Through *de novo* assembly, minicircle molecules with two conserved regions interspaced by two hypervariable regions 180° apart, were identified. Guide RNAs encoded within hypervariable regions were inferred and the mitochondrial mRNA editing cascades mediated by these guide RNAs were fully reconstructed. We also developed a PCR-based approach for the detection and confirmation of *T. lainsoni* kDNA using minicircle-specific primers. A deeper analysis on the diversity of minicircle hypervariable regions revealed that the three isolates correspond to different genotypes circulating in the region. This research significantly advances our understanding of the genomic architecture of *T. lainsoni* and offers valuable tools for its molecular identification, differentiation from other trypanosomes, and the study of kinetoplastid biology.

## Abstract (fr)

Le génome mitochondrial des Kinetoplastida, organisé en un réseau d'ADN unique appelé kinétoplaste, présente une complexité significative comparée à celle des autres génomes mitochondriaux eucaryotes. Les minicercles, qui constituent plus de 90 % de la masse du kinétoplaste, sont essentiels à l'édition post-transcriptionnelle des transcrits des maxicercles. Cette étude porte sur *Trypanosoma lainsoni*, un trypanosomatidé découvert initialement dans le nord du Brésil et signalé ultérieurement en Argentine. Grâce à une approche de séquençage combinée de deuxième et troisième génération, nous avons mené une analyse approfondie des minicercles d'ADNk de trois isolats argentins de *T. lainsoni*. Par assemblage *de novo*, des molécules de minicercles présentant deux régions conservées séparées par deux régions hypervariables diamétralement opposées (180°) ont été identifiées. Les ARN guides codés dans les régions hypervariables ont été identifiés et les cascades d'édition de l'ARNm mitochondrial qu'ils régulent ont été entièrement reconstituées. Nous avons également mis au point une méthode PCR pour la détection et la confirmation de l'ADNk de *T. lainsoni* à l'aide d'amorces spécifiques des minicercles. Une analyse plus approfondie de la diversité des régions hypervariables des minicercles a révélé que les trois isolats correspondent à différents génotypes circulant dans cette région. Ces travaux contribuent de manière significative à notre compréhension de l'architecture génomique de *T. lainsoni* et offrent des outils précieux pour son identification moléculaire, sa différenciation des autres trypanosomes et l'étude de la biologie des Kinetoplastida.

## Introduction

*Trypanosoma* (*Megatrypanum*) *lainsoni* Naiff & Barrett, 2013 is an apparently non-pathogenic kinetoplastid belonging to the subclass Metakinetoplastina, order Trypanosomatida, and family Trypanosomatidae [28]. It was first isolated from the rodent *Mesomys hispidus* (Rodentia, Echimyidae) in the State of Amazonas, northern Brazil, but was later identified in other mammals in the same country [29–31, 34, 39]. Our research group reported *T. lainsoni* in Argentina for the first time infecting *Leopardus geoffroyi* (Le29 isolate) and *Calomys* spp. rodents (Ca37 and Ca47 isolates) [16]. Despite these records, its epidemiologic significance remains unclear, and its distribution may be broader than currently recognized. The difficulties in culturing *T. lainsoni in vitro* represent a major challenge, hindering its biological and molecular characterization [28]. Although our research group recently reported the mitochondrial maxicircle genome of *T. lainsoni* [35], genomic information for this species remains extremely limited. This lack of information limits our understanding of its biology and the identification of potential molecular targets for PCR-based detection methods.

In this sense, our work focused on the minicircle component of the kinetoplastid mitochondrial genome (kDNA). kDNA is organized as a network of catenated minicircles and maxicircles [41]. Maxicircles are homologous to the mitochondrial DNA of other eukaryotes and encode several enzymes involved in the mitochondrial electron transport chain and ribosomal RNAs. Most protein-coding genes in maxicircles are encrypted and require post-transcriptional modification by editing the pre-mRNA through guide RNAs (gRNAs) encoded by minicircles [27]. The kDNA minicircles represent more than 90% of the kinetoplast mass. Depending on the species, these molecules can vary in size from 0.3 to 10 kb. Examples of this diversity include the minicircles of: *Leishmania braziliensis,* ~0.7 kb [9]; *T. cruzi,* ~1.4 kb [7, 9]*; T. brucei,* ~1 kb [11]; and *T. avium* notable for its ~10 kb minicircles, whose large size alters the shape of the kDNA disk [26]. At a structural level, minicircles have one or more conserved regions (CR) of 100–200 bp and one or more hypervariable regions (mHVRs). *Trypanosoma cruzi* parasites commonly have minicircles with four hypervariable regions interspersed with four conserved regions, while in *Leishmania* spp., minicircles have a single hypervariable region and a single conserved region [6, 44]. On the other hand, the minicircles of *T. brucei* also contain one conserved region, while those of *Crithidia fasciculata* contain two that are 180° apart. Despite minicircle size being constant within the same network, their gRNA-coding mHVRs are highly diverse [20, 38]. These diverse regions can encode for one to five different gRNAs, depending on the species, and even non-coding mHVRs can be found [11, 37, 42].

Due to their high copy number per cell, minicircles are interesting targets for DNA-amplification-based diagnosis methods [15, 38, 44, 50]. Primers for DNA amplification are mostly based on minicircle conserved regions. These structural components are characterized by the presence of three conserved sequence blocks (CSBs): CSB-1, CSB-2, and CSB-3 [33]. The CSB-1 block is a 10 bp sequence in which residues 1 and 10, and a main hexanucleotide sequence, are highly conserved in minicircles of all trypanosomatids. The CSB-2 block is an octanucleotide sequence in which six nucleotides are almost perfectly conserved in all trypanosomes. Lastly, CSB-3, also known as the Universal Minicircle Sequence (UMS), is a 12 bp block universally conserved in kinetoplastids and functionally represents the replication initiation site [22, 34]. Additionally, the heterogeneity of mHVRs has proven to be useful for trypanosome typing and species differentiation using methods such as LSSP-PCR [1, 17], qPCR [10], and PCR amplification followed by Sanger sequencing of amplicons [40] in *Leishmania* spp. Also, an NGS sequencing pipeline based on mHVRs has shown high resolution in the differentiation of the lineages and genotypes of *T. cruzi* [36, 38].

Genomic characterization is essential for identifying potential molecular targets for parasite detection, screening, and confirmation. Here, we characterized the structure of *T. lainsoni* kDNA minicircle molecules in Argentinian isolates, through deep sequencing and *de novo* assembly. Additionally, we explored the mRNA editing cascades mediated by guide RNAs, providing insights into the functionality of minicircles. Finally, a PCR-based approach using minicircle-derived primers was developed for the detection and confirmation of *T. lainsoni* kDNA.

## Materials and methods

### *Trypanosoma lainsoni* isolate culture and DNA extraction

The isolates were defrosted in biphasic medium composed of a solid phase (4% agar supplemented with rabbit blood) and a liquid phase composed of Liver Infusion Tryptose (LIT) medium supplemented with 20% fetal bovine serum (FBS), hemin 20 μg/mL, penicillin 100 IU, and streptomycin 100 μg/mL under shaking at 25 °C. Total DNA was extracted from the Le29, Ca37, and Ca47 *T. lainsoni* isolates. Total genomic DNA was extracted using a commercial kit following the manufacturer’s instructions (Quick-DNA MiniPrep, Zymo, Irvine, CA, USA). DNA quantification and quality assessment was performed using Nanodrop (Thermo Fisher Scientific, Waltham, MA, USA). DNA integrity was assessed through 0.8% agarose gel electrophoresis.

### Whole genome sequencing of *T. lainsoni*

The total DNA of isolates Le29, Ca37, and Ca47 was sequenced using an Illumina Novaseq 6000 platform (Novogene, Sacramento, CA, USA) with a paired end 2 × 150 bp kit. Additionally, the Le29 isolate was sequenced using the MinION Mk1C platform (Nanopore Technologies, ONT, Oxford, UK) after preparing the library with SQK-NBD112-24 kit (ONT). The library was run for 48 h in an R10.4 FLO-MIN112 (ONT) flow cell starting from 1 μg of total genomic DNA. Reads were deposited on Sequence Read Archive (SRA) and are available as part of the BioProject PRJNA1250468 (https://www.ncbi.nlm.nih.gov/bioproject/PRJNA1250468).

### Assembly of *T. lainsoni* minicircles from sequences obtained with Illumina technology

Illumina reads were evaluated using FastQC v0.12.1 (https://www.bioinformatics.babraham.ac.uk/projects/fastqc/), and then filtered and trimmed with Trimmomatic v0.32 [2] by applying the following parameters: LEADING:3 TRAILING:3 SLIDINGWINDOW:4:15 MINLEN:36. Pre-processed genomic reads from the three *T. lainsoni* isolates were used to assemble mitochondrial minicircles using the KOMICS bioinformatics pipeline [18, 48]. The assembly was carried out by running the “komics assemble” command with a list of 79, 99, and 119 *k-mers* allowing the extraction of possible linear minicircle contigs based on the presence of the highly conserved sequence block CSB-3 (GGGGTTG[G/A]TGTA). The *k-mer* size list was optimized to obtain a greater proportion of circularized minicircles in the assembly. After the assembly and identification of the minicircle contigs, the “komics circularize” and subsequently the “komics polish” commands were run by default to detect circular sequences, eliminate duplicates, and reorient the minicircles by placing CSB-1 at the beginning of each circularized contig. The quality of the assembly for each isolate was evaluated by mapping Illumina reads against assembled contigs using BWA-MEM2 v2.2.1 [49]. Mapping statistics, including the percentage of mapping reads, percentage of mapping reads containing CSB-3, and read depths were calculated using the KOMICS script “mapping_stats.sh”. Fully circularized minicircles were filtered based on their length and were then aligned using MEGA11 [43] to identify the conserved region containing CSB-1, CSB-2, and CSB-3 blocks.

To further validate the circularity of the assembled minicircle sequences, additional mapping was performed using Illumina reads. An in-house script was developed to extract the last 100 bases of each sequence and append them to the first 100 bases immediately after them to simulate a continuous circular structure. Illumina reads were next mapped against these modified sequences. If coverage was observed at the junction between the original last 100 bases and the appended first 100 bases, the sequence was confirmed to be circular. Coverage was visualized using IGV v2.16.2 [45].

### Identification of *T. lainsoni* minicircles in third generation sequencing reads

*Trypanosoma lainsoni* minicircles were identified from long-read whole-genome sequencing data of Le29, generated using the MinION ONT platform. A quality report of the reads was obtained using Nanoplot v1.41 [14], and reads were subsequently filtered with a minimum quality threshold of Q10 using Filtlong v0.2.1 (https://github.com/rrwick/Filtlong). Quality-filtered long reads were used to run a local nucleotide BLAST (blastn) search [8], using the CR of the assembled minicircles obtained in the previous step as query. Reads with more than 100 bp alignment against the CR and a *e*-value <0.05 were retained. The identified minicircles were filtered based on the most frequent length using a Python script, taking the length of KOMICS assembled minicircles as reference. To correct errors in these sequences, Illumina short reads were mapped to the minicircles identified in ONT long reads using BWA-MEM2 and three consecutive rounds of polishing were executed with Polypolish v0.5.0 [51] using the default parameters.

The polished Le29 minicircles identified in ONT reads were reoriented using Circlator 1.5.5 [21] command “circlator_fixstart” and the *T. lainsoni* CR as a query with ≥60% identity. These sequences were later aligned in MEGA to minicircle contigs previously obtained by the assembly of Illumina reads. Circularity was verified by inspecting the terminal alignments using MEGA.

### Validation of *T. lainsoni* minicircle structure by PCR

The primer design for the amplification of a *T. lainsoni* minicircle fragment was based on ~1,200 bp circularized minicircle sequences obtained using the KOMICS pipeline. Forward and reverse primers were designed to anneal to the CR, enabling full amplification of the hypervariable region. Primer selection was performed by visual inspection of aligned minicircle sequences, choosing ~20-nucleotide sequences that preferably included some of the CSBs. Several primer pairs were then tested *in silico* using the Primer3Plus web interface [46] to ensure they met the desired criteria, including melting temperature (with less than 1.5 °C difference between primers), GC content, length, and self-priming conditions for optimal annealing to the target. The primers were further validated by alignment in MEGA against all assembled minicircles, including those obtained by ONT sequencing. Primers were also tested through alignments using Primer-BLAST [52] against genomes stored in NCBI (https://www.ncbi.nlm.nih.gov/) of *T. cruzi, L. braziliensis*, and *L. infantum*, with negative results. The selected primer pair (LaimHVR_L: CGATACATGTTCCCCGTACAAT, Tm: 53 °C; LaimHVR_R: GCCCAAAATTTTGAACGCCC, Tm: 52 °C) was synthesized by GenBiotech (Buenos Aires, Argentina).

The optimal annealing temperature, primer concentrations, and template DNA dilutions were standardized to ensure the correct amplification of mHVRs in *T. lainsoni* isolates. *Trypanosoma cruzi* and *Leishmania* spp. DNA samples were included to assess specificity. PCR amplifications were performed in a Veriti thermocycler (Applied Biosystems, Foster City, CA, USA) in a final volume of 50 μL, using 0.3 μM forward and reverse primers and 0.1 ng of DNA. Reactions followed the GoTaq G2 DNA polymerase protocol (Promega Corporation, Madison, WI, USA) with the following cycling conditions: initial denaturation at 95 °C for 120 s, followed by 30 cycles of 30 s at 95 °C, 30 s at 50 °C, and 60 s at 72 °C, with a final extension at 72 °C for 300 s. Amplification products were analyzed by 1.5% agarose gel electrophoresis at 90 V for ~45 min.

### Diversity analysis of minicircle hypervariable regions of *T. lainsoni*

Illumina paired-end reads from each *T. lainsoni* isolate were mapped against a reference file containing all mHVRs obtained from the genome assembly using BWA-MEM2 v2.2.1, and BAM files were filtered to retain mapped reads. Sorted and indexed BAM files were generated using Samtools v1.21 [13], and per-base coverage (.bedgraph) files were produced with Bedtools v2.31.1 [32]. An in-house Python script processed .bedgraph files by filtering positions with coverage ≥20 reads, retaining mHVRs with ≥340 covered bases, calculating median coverage per mHVR, and exporting results to .tsv files. Coverage tables from all isolates were merged into a table of mHVRs relative abundances using a Python algorithm. This table was imported into QIIME 2 v2024.10 [3] for diversity analysis. To ensure comparability across samples, rarefaction was applied to standardize sequencing depth prior to diversity estimation. Core diversity metrics were computed to provide insight into community structure differences among isolates. Diversity metrics included the observed features index as an indicator of alpha-diversity and Bray-Curtis dissimilarity for beta-diversity. A Principal Coordinate Analysis (PCoA) plot was generated from the Bray-Curtis dissimilarity matrix.

### Guide RNA identification

Guide RNAs (gRNAs) located within the mHVRs of *T. lainsoni*, which guide the editing of maxicircle-encoded mRNAs, were identified using the algorithm described by Rusman *et al.* 2021 [37]. Edited mRNAs corresponding to *ATP synthase subunit 6* (*ATPase 6*), *cytochrome c oxidase subunit III* (*COIII*), *C-rich region 3* (*CR3*) with unknown-function (also called *G-rich region 3, G3*), *C-rich region 4* (*CR4*) with unknown-function (also called *G-rich region 4, G4*) [23], *NADH dehydrogenase subunit 3* (*ND3*), *NADH dehydrogenase subunit 7* (*ND7*), *NADH dehydrogenase subunit 8* (*ND8*), *NADH dehydrogenase subunit 9* (*ND9*), and *ribosomal protein S12* (*RPS12*) were predicted based on *T. lainsoni* maxicircle DNA sequences [35] and following the methodology described by Rusman *et al.* [37]. A gRNA class was defined as a group of gRNAs targeting the same mRNA region. Editing cascades were reconstructed and visualized for each *T. lainsoni* isolate. The gRNA position within the mHVRs was determined as the region where at least one gRNA was mapped using the algorithm proposed by Rusman *et al.* [37].

## Results

### Structure of *T. lainsoni* minicircles

**Figure 1:**
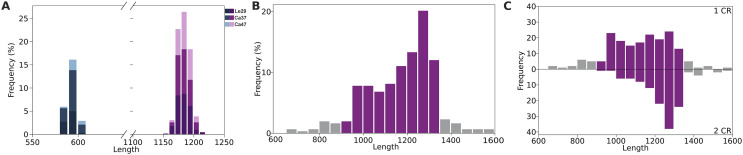
Length frequency histograms of *Trypanosoma lainsoni* minicircles. A) KOMICS-circularized contigs were grouped into two categories based on their length (in base pairs): short contigs (blue shades, 25%) and long contigs (purple shades, 75%). Dark bars represent Le29 isolate, intermediate shades represent Ca37, and light shades represent Ca47. B) ONT reads filtered by BLAST alignment to the minicircle CR. The most frequent read lengths (600–1,600 bp) are shown. Reads between 900–1,300 bp, representing 77.3% of filtered reads, are highlighted in purple. C) Length frequency histograms of ONT-derived minicircles grouped by the number of CRs. The upper panel shows minicircles with a single CR; 79.0% of these falls within the 900–1,300 bp range (highlighted in purple). The lower panel shows minicircles with two CRs; 74.8% fall within the same range.

**Supplementary Figure 1:**
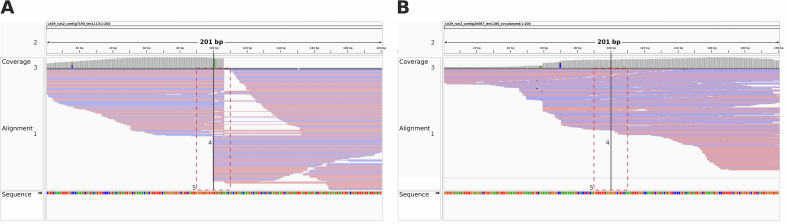
Circularization analysis of minicircle sequences of Le29 isolate obtained through mapping and visualization in IGV. The compressed graphs show the alignment between paired-end Illumina reads (1), represented as horizontal lines, and the merged sequences (2), displayed along a 201 bp axis. Merged sequences were obtained by extracting the last 100 bases of each sequence and appending them to the beginning of the first 100 bases of such sequences. The bars represent coverage at each position of the axis (3). Red lines indicate forward-strand reads, and blue lines indicate reverse-strand reads. A vertical line (4) marks the junction where the ends merge, and the box (5) highlights the region where overlap is expected to verify circularity. A) Coverage of Illumina reads in a non-circularized contig. Moderate coverage is observed at the junction region, and no reads were found to be shared between both ends. B) Alignment of Illumina reads against correctly circularized contigs shows high coverage at the junction position of the merged sequence, with reads shared between both ends.

*De novo* assembly of minicircles using Illumina reads generated 2,654 linear contigs containing CSB-3, of which 638 were classified as circularized by KOMICS (Supplementary Table 1). On average, 8.22% of the total Illumina reads from each of the three isolates were mapped to minicircle contigs. Relatively high read depths were observed in all contigs, suggesting robust coverage and high accuracy of the obtained sequences. The mapping statistics are summarized in Supplementary Table 2. Based on length frequencies of the assembled sequences, 2,476 contigs smaller than 1,600 bp were retained. Alignment of the assembled minicircles allowed for the identification of a consensus sequence of approximately 138 bp, representing the CR of *T. lainsoni* minicircles which includes the three conserved blocks: CSB-1, CSB-2, and CSB-3. The size-filtered contigs were then classified into short contigs (582–607 bp, mean 593.6 bp) showing one CR, and long contigs (1,157–1,213 bp, mean 1,184.4 bp) showing two CRs interspaced by two hypervariable regions of 441–469 bp (Fig. 1A, Supplementary Table 3). Minicircle contigs with two CRs were the most frequent. In addition, Illumina reads mapped to the merged ends of these contigs showed high coverage in the junction region, which is consistent with a continuous circular sequence (Supplementary Figure 1).

In addition, complete minicircle sequences were identified in ONT reads through a BLAST search using the CR sequence identified in Illumina contigs as a query. A total of 535 alignments with identities ranging from 82.4% to 100% were obtained. These alignments correspond to 348 reads with lengths between 296 bp and 40 kb. The most frequent lengths were retained, ranging from 600 to 1,600 bp, thereby removing potential sequencing artifacts. A total of 308 sequences were obtained, of which 77.3% were between 900 and 1,300 bp (Fig. 1B). A total of 127 minicircles contained two CRs, whereas 181 contained only one CR (Fig. 1C, Table 1). Sequences with a single CR were further analyzed using BLAST to identify a second CR that might not have been fully aligned. The presence of at least one of the three CSBs was detected in 25 minicircles between positions 600–800. Furthermore, a comparison of length distributions between these two groups of minicircles showed that those with two conserved regions ranged from 944 to 1,558 bp, whereas those with a single conserved region ranged from 676 to 1,595 bp (Fig. 1C)**.**

**Table 1:** Minicircle sequences identified in ONT sequences by BLAST.

No. of CRs	Number of sequences	Mean length	Median length	SD
1	181	1,125.2	1,138	165.30
2	127	1,244.4	1,274	107.90
Total	308	1,174.4	1,209	155.74

Illumina reads were mapped against the minicircle ONT sequences to correct errors. Three consecutive rounds of polishing were executed with Polypolish. On average, the length of the sequences before and after polishing was 1,174.40 bp and 1,181.1 bp, respectively. This last length was close to the average length of 1,184.4 bp of long contigs assembled using Illumina reads. There were no modifications at the ends of the minicircle sequences due to a low sequencing depth in these regions. These observations would confirm the length of the *T. lainsoni* minicircles to be around 1,184 bp and that short contigs obtained with KOMICS were assembly artifacts. The circularity of ONT minicircle sequences was assessed by alignment to the circularized minicircles assembled by KOMICS, confirming the correct alignment of CRs.

### Minicircle Conserved Region sequence analysis

**Figure 2:**
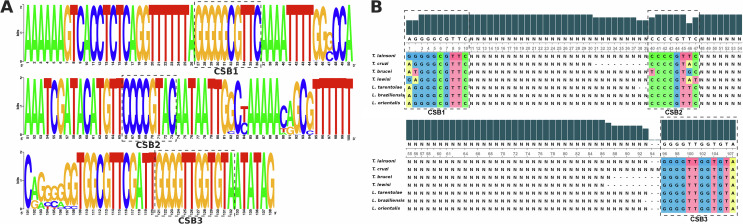
Conserved Region alignments. A) Sequence logo of *Trypanosoma lainsoni* kDNA minicircle conserved region. Nucleotide frequency in each position of a multiple alignment of minicircle assembled sequences is shown. Each base is represented by its corresponding letter and the height of each symbol represents its frequency. Dashed lines indicate CSB-1, CSB-2, and CSB-3 conserved blocks. B) Sequence comparison of conserved blocks shared by different trypanosomatids species. CSB-1, CSB-2, and CSB-3 blocks in *T. lainsoni* minicircles are highly conserved against the trypanosomatids conserved blocks consensus sequence. However, CSB-1 presents a substitution at the first position (A → G). The CSB-1/CSB-2 distance is conserved in *T. lainsoni* with respect to *T. brucei*, *T. lewisi*, *L. braziliensis*, and *L. orientalis*, while the CSB-2/CSB-3 distance is variable, being similar to *Leishmania* and two nucleotides shorter than in *T. cruzi*.

**Supplementary Figure 2:**
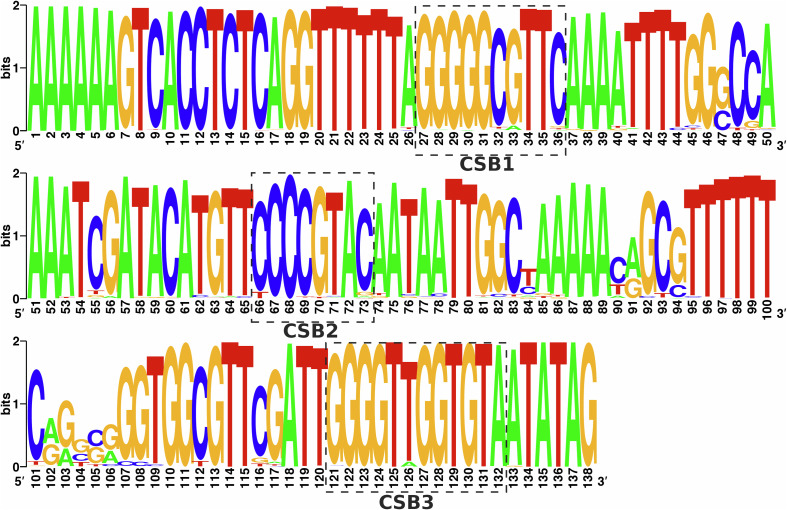
Sequence Logo represents the conserved region of minicircle sequences of T. lainsoni obtained by ONT. The graph shows the frequency of nucleotides in each minicircles multiple alignment position. Dotted lines mark conserved blocks CSB-1, CSB-2, and CSB-3. Similarly to minicircle contigs obtained with Illumina sequencing, these blocks show a high conservation level despite the higher error rate during ONT base calling.

Consensus CR sequences (~138 bp) from KOMICS-assembled and ONT-derived minicircles of *T. lainsoni* are shown in Figure 2A and Supplementary Figure 2, respectively. The three conserved blocks: CSB-1, CSB-2, and CSB-3 were identified and showed no variations among the three isolates. The conserved blocks of *T. lainsoni* were highly similar to those of other trypanosomatids. A substitution (A → G) was observed at the first position of CSB-1 in *T. lainsoni*. This substitution was shared with *T. lewisi,* although this species showed an additional substitution at the second position of CSB-1 (G → A). In contrast, the CSB-2 sequence of *T. lainsoni* was conserved relative to *Leishmania* spp., while one and two substitutions were observed compared with the CSB-2 sequences of *T. cruzi* and *T. lewisi/T. brucei*, respectively. The distance between CSB-2 and CSB-3 was shorter than in *T. cruzi*, but longer than in other trypanosomatids (Fig. 2B). As expected, CSB-3 was perfectly conserved in the *T. lainsoni* sequence.

### Validation and detection of *T. lainsoni* minicircle by PCR

**Figure 3:**
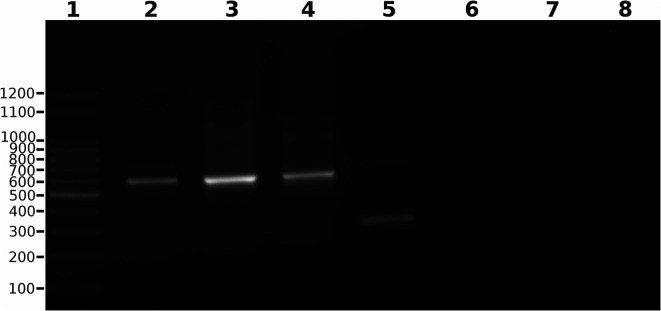
1.5% Agarose gel stained with ethidium bromide showing PCR amplification products using primers designed for *Trypanosoma lainsoni* mHVRs. Lanes: 1) 100 bp molecular weight marker, 2) *T. lainsoni* Le29, 3) *T. lainsoni* Ca37, 4) *T. lainsoni* Ca47, 5) *T. cruzi*, 6) *L. braziliensis*, 7) *L. infantum*, and 8) Blank.

**Figure 4:**
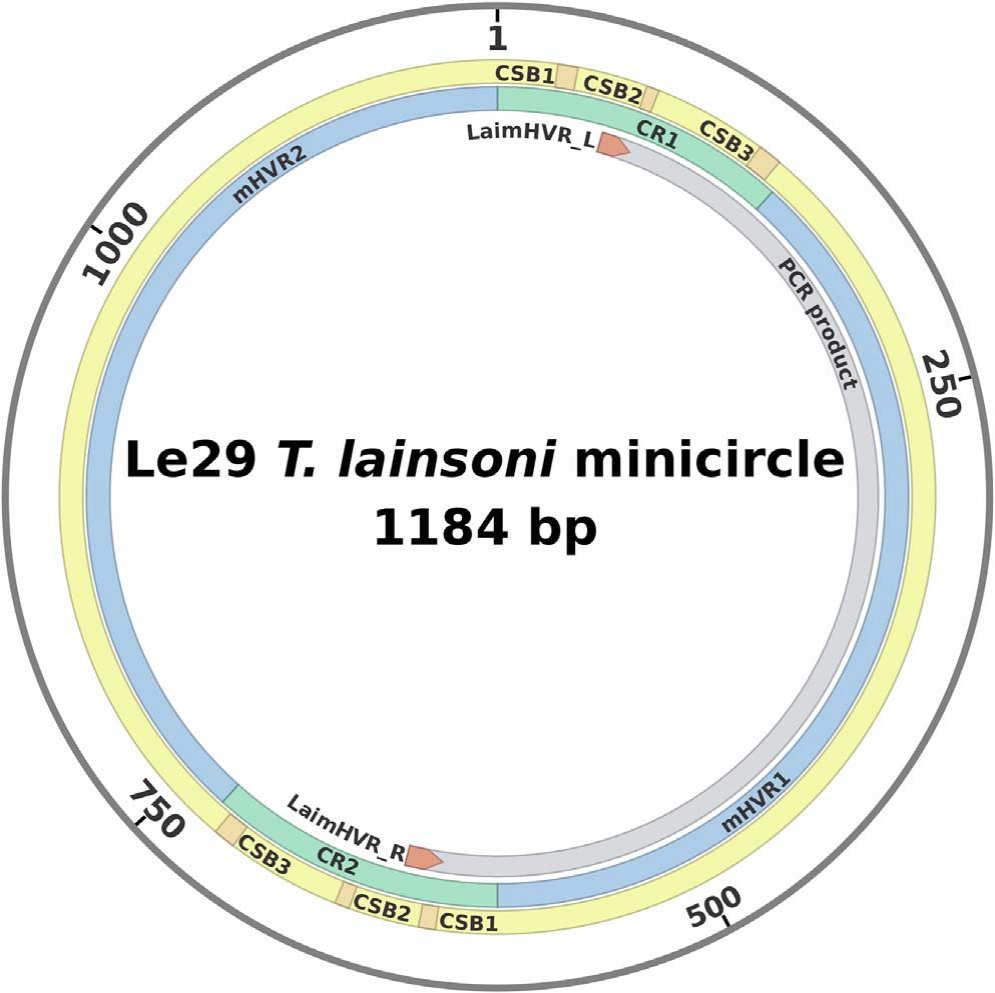
Schematic representation of the *Trypanosoma lainsoni* minicircle (1,184 bp) from Le29 isolate. The outermost ring (yellow) highlights the positions of the conserved sequence blocks (CSB-1, CSB-2, and CSB-3) in each CR (green segments). The inner ring (grey) shows the expected PCR amplification product obtained with the primers LaimHVR_L and LaimHVR_R (red), which bind to the conserved regions flanking the mHVRs (blue segments).

An amplification product of the expected size (~580 bp) was obtained from the three *T. lainsoni* isolates by using LaimMHVR_L and LaimHVR_R primers (Fig. 3). No amplification was observed in the specificity control with *L. braziliensis* and *L. infantum* isolates. However, a ~ 350 bp product was detected in the *T. cruzi* PCR reaction. The PCR results empirically confirmed the length of the conserved and hypervariable regions of *T. lainsoni* minicircles, as described in previous sections based on the combined analysis of second- and third-generation sequencing data. Figure 4 illustrates the inferred structure of the *T. lainsoni* minicircle and the annealing positions of the primers in the conserved regions.

### Diversity analysis of minicircles hypervariable regions of *T. lainsoni*

**Figure 5:**
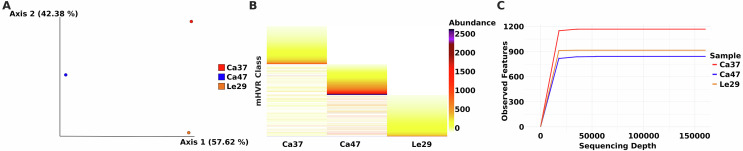
Beta diversity and abundance of mHVRs among *Trypanosoma lainsoni* isolates. A) Principal Coordinate Analysis (PCoA) based on Bray–Curtis dissimilarity, illustrating beta diversity among *T. lainsoni* isolates. The plot shows three distinct clusters (Ca37, Ca47, and Le29), indicating clear differences in composition and abundance of mHVRs. The separation along Axis 1 (57.62%) and Axis 2 (42.38%) suggests significant dissimilarities in genetic patterns among isolates. B) Heatmap showing the abundance and distribution of mHVR classes across isolates. The *y*-axis represents different mHVR classes, while the *x*-axis corresponds to the isolates. The abundance of sequences in each mHVR class is represented using a gradient color scale. C) Rarefaction curves showing the number of mHVR classes as a function of sequencing depth for *T. lainsoni* isolates (Ca37, Ca47, and Le29). The curves reach a plateau, indicating that sequencing depth was sufficient to capture the total diversity of each sample. Differences in alpha diversity among isolates suggest variations in richness of mHVR profiles.

Based on coverage analysis of Illumina reads mapped against the 2,440 assembled mHVR sequences (mHVR classes), the following relative abundances of mHVRs were obtained for each isolate: 256,864 for Ca37, 516,961 for Ca47, and 160,080 for Le29. Principal Coordinate Analysis (PCoA) based on Bray–Curtis dissimilarity was performed to visualize beta diversity among samples. The PCoA plot (Fig. 5A) clearly differentiates the three isolates based on their mHVR composition. This pattern was further supported by the heatmap analysis (Fig. 5B), which showed that the composition of mHVR classes is largely unique to each isolate, with only a few shared classes among them: 38 out of 2440 mHVR classes were present in all isolates. The limited overlap in mHVR classes suggests a high degree of genetic differentiation, aligning with the observed distribution of isolates in the PCoA plot.

Alpha diversity analysis was conducted to assess richness, and rarefaction curves indicated that sequencing depth was sufficient to capture the total diversity of mHVRs classes, as all curves reached a plateau (Fig. 5C). These results highlight notable dissimilarities among samples based on their mHVR profiles and confirm that sequencing depth was adequate to represent mHVR diversity, further supporting the presence of distinct genetic patterns among *T. lainsoni* isolates.

### *Trypanosoma lainsoni* mHVRs encode complete gRNA repertories

**Figure 6:**
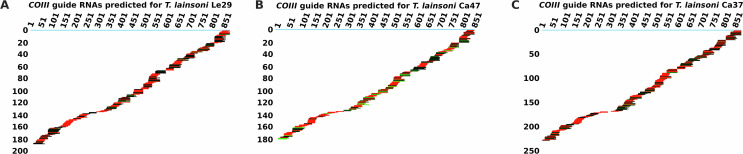
Editing cascades of COIII mRNA, predicted from the sequencing reads of each *Trypanosoma lainsoni* isolate: A) Le29, B) Ca47, and C) Ca37. Guide RNAs are shown aligned below the COIII mRNA (light blue line) according to their editing position. The *x*-axis represents the mRNA position, while the *y*-axis shows the cumulative number of gRNA classes up to that position. gRNA classes are color-coded according to sequence abundance as follows: red (1–19 reads), black to green (20–1,000 reads), and light green (>1,000 reads).

**Supplementary Figure 3:**
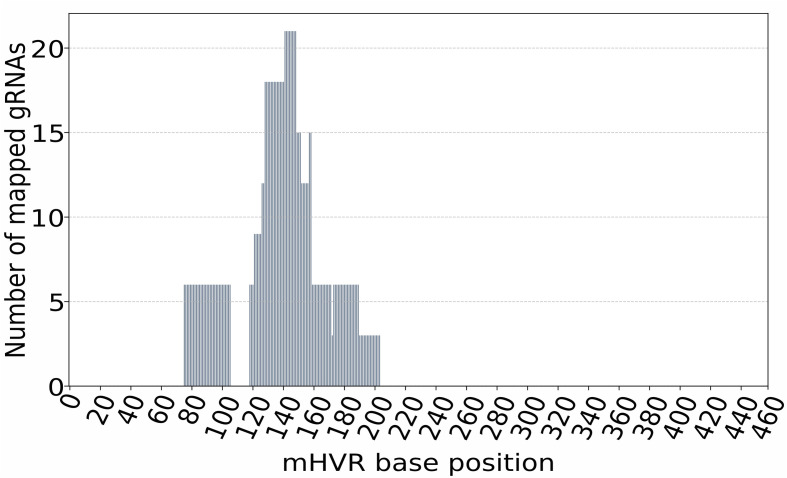
Mapping of estimated guide RNA (gRNA) sequences to the hypervariable region (mHVR) of T. lainsoni minicircles. The number of estimated gRNAs mapped to each base position in the mHVR is shown. The distribution indicates that the estimated positions of gRNAs range from approximately 77 bp to 202 bp downstream of CSB-3.

Editing cascades were reconstructed for the nine genes whose edited mRNA sequences were previously predicted. Editing cascades for *COIII*, a pan-edited gene, and the remaining mRNAs are shown in Figure 6 and Supplementary File 1, respectively. Between 1,161 and 1,449 gRNA classes were predicted for each isolate. The number of gRNA classes found for each edited mRNA is summarized in Supplementary Table 4. Nearly complete editing cascades were obtained for all genes. Additionally, the estimated position of the guide RNA in the hypervariable region of the minicircle was found between 77 bp and 202 bp downstream of CSB-3 (Supplementary Figure 3).

## Discussion

Due to its recent discovery, several biological aspects of the kinetoplastid protozoan *T. lainsoni* remain unknown. So far, this species has been found to parasitize a wide variety of flying and terrestrial mammals [16, 28–31, 34, 39]. However, key aspects such as its life cycle, vectors, pathogenicity, and potential to cause diseases in humans or other animals remain undescribed. In this context, the availability of mitochondrial genomic data for this organism could provide valuable insights into its biology and diversification. This study represents a significant step forward in understanding the mitochondrial genomic organization of *T. lainsoni*, shedding light on the structural and functional complexity of its minicircles. By integrating second- and third-generation sequencing, we successfully assembled and characterized minicircle sequences from three Argentinian isolates (Le29, Ca37, and Ca47), describing their architecture with two hypervariable regions interspaced by conserved regions.

In the first stage, minicircles were assembled using the KOMICS bioinformatics pipeline. Assembled sequences with atypical lengths (6.71%) were observed, which could have originated from the nuclear genome or maxicircles. As no reference genomes were available, the origin of these sequences could not be determined; therefore, a cut-off criterion of 1,600 bp was established based on the most frequent lengths to ensure the exclusion of assembly artifacts. However, since the structure of assembled contigs was expected to be circular, sequences were filtered to retain only those that were fully circularized. Most (75%) of the circularized contigs recovered were sequences between 1,157 and 1,213 bp (mean: 1,184.4 bp) and the rest were short circularized contigs between 580 and 620 bp. Alignment analysis of the assembled minicircle contigs enabled the identification of the conserved region (138 bp) containing its three characteristic conserved sequence blocks (CSB-1, CSB-2, and CSB-3). The ultrastructure of the minicircles was further confirmed by identifying full-length minicircles in third-generation sequencing data from the Le29 isolate, using the previously identified CR sequence as a query. On average, the length of the filtered sequences before polishing was 1,174 bp, while after polishing, it was 1,181 bp. This result confirmed that short minicircle contigs were likely artifacts from the assembly process and supported the mean length (~1,184 bp) obtained from the Illumina-based assemblies. Additionally, the presence of two conserved regions interspersed with two hypervariable regions was corroborated by third-generation sequencing data. The circularity of these molecules was thoroughly verified by aligning the circularized molecules obtained with KOMICS against the minicircles identified in ONT reads, along with additional circularity verification using a mapping strategy. The minicircle size and structure were validated by PCR, which yielded a single amplicon of the expected size for all three isolates.

Minicircle structural arrangement varies among trypanosomatids: in *Leishmania* spp. and *T. brucei*, minicircles contain a single CR [5, 12, 24, 42], whereas in *T. cruzi*, minicircles typically contain four CRs arranged at ~90° intervals [7, 19, 25]. Other species, such as *T. rangeli* and *T. copemani*, exhibit variable numbers of CRs [4, 47]. Here, we describe *T. lainsoni* minicircles with two hypervariable regions (mHVRs) with an average length of 454 bp. The alignment of circularized, non-circularized, and long reads resulted in the confirmation of the CR–mHVR–CR–mHVR pattern for minicircles sequences. The two mHVRs exhibited similar sizes and maintained a consistent separation between conserved regions, resulting in a symmetrical minicircle structure, with conserved regions positioned approximately 180° apart. This structural organization is similar to that of *T. lewisi* minicircles, which also contain two CRs separated by ~180° [25]. An interesting observation in ONT-derived minicircle sequences was that 181 sequences contained a single CR, whereas 127 had two CRs. Sequences with two CRs (944–1,558 bp) showed a more homogeneous length distribution (*σ* = 107.9) than those with one CR (676–1,595 bp, *σ* = 163.3). A deeper analysis of minicircle sequences with a single CR revealed that at least 25 sequences contained additional CSBs, suggesting the presence of an incomplete second CR. The higher error rate associated with ONT sequencing could partly explain why a completely conserved region could not be detected in some cases. However, the presence of minicircles with a single conserved region cannot be ruled out. If such minicircles exist, it is unlikely that they would be detected as amplicons with larger hypervariable regions, since the PCR reaction designed here would probably favor the amplification of the ~450 bp mHVR region located between the primers. Further studies are required to confirm the existence of minicircles containing only one conserved region.

Importantly, diversity analyses of mHVRs from the three isolates revealed high dissimilarity, suggesting the presence of distinct genotypes circulating in the study area, potentially aiding epidemiologic surveillance and ecological studies. Deep sequencing of mHVRs and cluster analysis have previously been proposed as tools for lineage and genotype identification in *T. cruzi* [38]. Similar differences have also been observed in guide RNA (gRNA) sequences, suggesting a link between genotype and minicircle functionality in different *T. cruzi* lineages [37].

In this study, we reconstructed the complete editing cascades of mRNAs guided by minicircle encoded gRNAs. Unlike other trypanosomatids, such as *T. cruzi* and *L. tarentolae*, where incomplete editing cascades have been reported for some genes of the respiratory complex I [37, 42], *T. lainsoni* appears to retain a fully functional editing system for all the studied genes. This suggests that the edited mRNAs encode functional enzymes, which may have implications for parasite metabolism and adaptation. Further studies are needed to determine whether this complete editing cascade is a conserved feature among different *T. lainsoni* isolates and how it influences mitochondrial function.

Finally, *T. lainsoni* minicircle sequences obtained in this study were used to develop a PCR assay for detecting this parasite in DNA samples. This assay allowed empirical testing of the expected size of the mHVRs. Amplification of a ~ 580 bp product was observed in the three available isolates of *T. lainsoni*, which allowed differentiation of this species from *L. braziliensis* and *L. infantum*, where no amplification was observed. However, a band corresponding to a *T. cruzi* minicircle fragment of approximately 350 bp was observed, which was smaller size than that obtained for *T. lainsoni*. A detailed comparative analysis of the CR with those from other trypanosomatids revealed that, as expected, CSB-2 and CSB-3 were highly conserved relative to a consensus sequence. In contrast, CSB-1 was only partially conserved, with an A → G substitution at position 1 compared to the CSB-1 block in other trypanosomatids. Although *in silico* specificity testing was performed, PCR primers were designed to target the conserved CSB-1 and CSB-2 blocks. Therefore, it is not surprising that they also amplified *T. cruzi* minicircles. However, the resulting amplicons were easily distinguishable by size, as the *T. cruzi* fragment (~350 bp) was notably smaller than the *T. lainsoni* product (~580 bp). This work lays the groundwork for further studies to optimize and validate this PCR assay, including a more comprehensive evaluation of its sensitivity and specificity using additional trypanosomatid species, *T. lainsoni* isolates, and biological samples obtained from field studies. Therefore, the assay presented here should be considered a preliminary approach for the molecular detection of *T. lainsoni*. Future research should focus on the biological and epidemiologic significance of *T. lainsoni*, and its potential hosts and vectors. The findings in this work not only expand our knowledge of *T. lainsoni* genetics but also contribute to the development of molecular diagnostic tools with implications for parasite surveillance.

## Conclusions

In this study, we successfully assembled and accurately described the complete structure of *T. lainsoni* minicircles by combining second- and third-generation sequencing technologies. The *T. lainsoni* minicircles consist of circular DNA molecules with an average length of 1,184 bp, containing two highly conserved regions of 138 bp positioned approximately 180° apart and separated by two hypervariable regions of ~454 bp each. The conserved regions harbor the canonical CSB1, CSB2, and CSB3 sequence blocks, which were found to be highly conserved among the three isolates, as well as in comparison to other trypanosomatids. The structural arrangement of the minicircles was further supported through the development of a preliminary PCR-based assay, which enabled the detection of the parasite in DNA samples. Although additional optimization and validation studies are still required to assess its sensitivity and specificity, this approach represents a promising tool for the future identification of potential hosts and vectors. Additionally, the minicircle-derived sequences were used to reconstruct complete mRNA editing cascades guided by minicircle-encoded gRNAs. These analyses suggested that *T. lainsoni* retains a fully functional editing system for all studied genes, potentially linked to conserved mitochondrial function and to parasite metabolic adaptation. The diversity analysis of mHVRs revealed considerable sequence heterogeneity among the isolates, suggesting that the method was effective in capturing the complexity of minicircle variation and could be applied to differentiate among circulating genotypes. This work contributes valuable information to our understanding of *T. lainsoni* mitochondrial genomics and establishes a basis for future studies exploring its biology, epidemiology, and the development of molecular tools for surveillance.

## Supplementary material

*Supplementary File 1*: Editing cascades of ATPase 6, CR3, CR4, ND3, ND7, ND8, ND9, and RPS12 mRNAs, predicted from the sequencing reads of each T. lainsoni isolate: Le29, Ca47, and Ca37. Guide RNAs are shown aligned below the mRNA sequences according to their editing position. The x-axis represents the mRNA position, while the y-axis shows the cumulative number of gRNA classes up to that position. gRNA classes are color-coded according to sequence abundance as follows: red (1–19 reads), black to green (20–1,000 reads), and light green (>1,000 reads). *Supplementary Table 1*: Summary of contigs obtained by KOMICS pipeline. *Supplementary Table 2*: Mapping statistics of Illumina reads against contigs obtained by KOMICS. *Supplementary Table 3*: Summary of size filtered *T. lainsoni* minicircle contigs. The supplementary material of this article is available at https://www.parasite-journal.org/10.1051/parasite/2026034/olm.

## References

[1] Alvarenga JSC, Ligeiro CM, Gontijo CMF, Cortes S, Campino L, Vago AR, Melo MN. 2012. KDNA genetic signatures obtained by LSSP-PCR analysis of *Leishmania (Leishmania) infantum* isolated from the new and the old world. PLoS One, 7, e43363. 22912862 10.1371/journal.pone.0043363 PMC3422226

[2] Bolger AM, Lohse M, Usadel B. 2014. Trimmomatic: A flexible trimmer for Illumina sequence data. Bioinformatics, 30, 2114–2120. 24695404 10.1093/bioinformatics/btu170 PMC4103590

[3] Bolyen E, Rideout JR, Dillon MR, Bokulich NA, Abnet CC, Al-Ghalith GA, Alexander H, Alm EJ, Arumugam M, Asnicar F, Bai Y, Bisanz JE, Bittinger K, Brejnrod A, Brislawn CJ, Brown CT, Callahan BJ, Caraballo-Rodríguez AM, Chase J, Cope EK, Da Silva R, Diener C, Dorrestein PC, Douglas GM, Durall DM, Duvallet C, Edwardson CF, Ernst M, Estaki M, Fouquier J, Gauglitz JM, Gibbons SM, Gibson DL, Gonzalez A, Gorlick K, Guo J, Hillmann B, Holmes S, Holste H, Huttenhower C, Huttley GA, Janssen S, Jarmusch AK, Jiang L, Kaehler BD, Kang KB, Keefe CR, Keim P, Kelley ST, Knights D, Koester I, Kosciolek T, Kreps J, Langille MGI, Lee J, Ley R, Liu Y-X, Loftfield E, Lozupone C, Maher M, Marotz C, Martin BD, McDonald D, McIver LJ, Melnik AV, Metcalf JL, Morgan SC, Morton JT, Naimey AT, Navas-Molina JA, Nothias LF, Orchanian SB, Pearson T, Peoples SL, Petras D, Preuss ML, Pruesse E, Rasmussen LB, Rivers A, Robeson MS, Rosenthal P, Segata N, Shaffer M, Shiffer A, Sinha R, Song SJ, Spear JR, Swafford AD, Thompson LR, Torres PJ, Trinh P, Tripathi A, Turnbaugh PJ, Ul-Hasan S, Hooft JJJ van der, Vargas F, Vázquez-Baeza Y, Vogtmann E, Caporaso JG. 2019. Reproducible, interactive, scalable and extensible microbiome data science using QIIME 2. Nature Biotechnology, 37, 852–857. 10.1038/s41587-019-0209-9 PMC7015180 31341288

[4] Botero A, Kapeller I, Cooper C, Clode PL, Shlomai J, Thompson RCA. 2018. The kinetoplast DNA of the Australian trypanosome, *Trypanosoma copemani*, shares features with *Trypanosoma cruzi* and *Trypanosoma lewisi*. International Journal for Parasitology, 48, 691–700. 29778329 10.1016/j.ijpara.2018.02.006

[5] Brewster S, Barker DC. 2002. Analysis of minicircle classes in *Leishmania* (*Viannia*) species. Transactions of the Royal Society of Tropical Medicine and Hygiene, 96 Suppl 1, S55–63. 12055852 10.1016/s0035-9203(02)90052-0

[6] Bruijn MH de, Barker DC. 1992. Diagnosis of New World leishmaniasis: specific detection of species of the *Leishmania braziliensis* complex by amplification of kinetoplast DNA. Acta Tropica, 52, 45–58. 1359760 10.1016/0001-706x(92)90006-j

[7] Callejas-Hernández F, Herreros-Cabello A, Del Moral-Salmoral J, Fresno M, Gironès N. 2021. The complete mitochondrial DNA of *Trypanosoma cruzi*: Maxicircles and minicircles. Frontiers in Cellular and Infection Microbiology, 11, 672448. 34268138 10.3389/fcimb.2021.672448 PMC8277381

[8] Camacho C, Coulouris G, Avagyan V, Ma N, Papadopoulos J, Bealer K, Madden TL. 2009. BLAST+: architecture and applications. BMC Bioinformatics, 10, 421. 20003500 10.1186/1471-2105-10-421 PMC2803857

[9] Camacho E, Rastrojo A, Sanchiz Á, González-de la Fuente S, Aguado B, Requena JM. 2019. *Leishmania* mitochondrial genomes: maxicircle structure and heterogeneity of minicircles. Genes, 10, 758. 31561572 10.3390/genes10100758 PMC6826401

[10] Ceccarelli M, Buffi G, Diotallevi A, Andreoni F, Bencardino D, Vitale F, Castelli G, Bruno F, Magnani M, Galluzzi L. 2020. Evaluation of a kDNA-Based qPCR assay for the detection and quantification of Old World *Leishmania* species. Microorganisms, 8, 2006. 33339158 10.3390/microorganisms8122006 PMC7765608

[11] Cooper S, Wadsworth ES, Ochsenreiter T, Ivens A, Savill NJ, Schnaufer A. 2019. Assembly and annotation of the mitochondrial minicircle genome of a differentiation-competent strain of *Trypanosoma brucei*. Nucleic Acids Research, 47, 11304–11325. 31665448 10.1093/nar/gkz928 PMC6868439

[12] Cooper S, Wadsworth ES, Schnaufer A, Savill NJ. 2022. Organization of minicircle cassettes and guide RNA genes in *Trypanosoma brucei*. RNA (New York), 28, 972–992. 10.1261/rna.079022.121 PMC9202587 35414587

[13] Danecek P, Bonfield JK, Liddle J, Marshall J, Ohan V, Pollard MO, Whitwham A, Keane T, McCarthy SA, Davies RM, Li H. 2021. Twelve years of SAMtools and BCFtools. GigaScience, 10, giab008. 33590861 10.1093/gigascience/giab008 PMC7931819

[14] De Coster W, D’Hert S, Schultz DT, Cruts M, Van Broeckhoven C. 2018. NanoPack: visualizing and processing long-read sequencing data. Bioinformatics, 34, 2666–2669. 29547981 10.1093/bioinformatics/bty149 PMC6061794

[15] Degrave W, Fragoso SP, Britto C, Heuverswyn H van, Kidane GZ, Cardoso MA, Mueller RU, Simpson L, Morel CM. 1988. Peculiar sequence organization of kinetoplast DNA minicircles from *Trypanosoma cruzi*. Molecular and Biochemical Parasitology, 27, 63–70. 2830509 10.1016/0166-6851(88)90025-4

[16] Díaz AG, Ragone PG, Rusman F, Floridia-Yapur N, Barquez RM, Díaz MM, Tomasini N, Diosque P. 2020. A novel genotype and first record of *Trypanosoma lainsoni* in Argentina. Pathogens, 9, 731. 32899895 10.3390/pathogens9090731 PMC7558950

[17] Ferreira GA, Soares FCS, Vasconcellos SA, Rodrigues EHG, Werkhäuser RP, Brito MEF de, Abath FGC. 2007. Discrimination of *Leishmania braziliensis* variants by kDNA signatures produced by LSSP-PCR. Journal of Parasitology, 93, 712–714. 17626371 10.1645/GE-958R1.1

[18] Geerts M, Schnaufer A, Van den Broeck F. 2021. rKOMICS: an R package for processing mitochondrial minicircle assemblies in population-scale genome projects. BMC Bioinformatics, 22, 468. 34583651 10.1186/s12859-021-04384-1 PMC8479924

[19] Gómez-Palacio A, Cruz-Saavedra L, Van den Broeck F, Geerts M, Pita S, Vallejo GA, Carranza C, Ramírez JD. 2024. High-throughput analysis of the *Trypanosoma cruzi* minicirculome (mcDNA) unveils structural variation and functional diversity. Scientific Reports, 14, 5578. 38448494 10.1038/s41598-024-56076-4 PMC10917808

[20] Hong M, Simpson L. 2003. Genomic organization of *Trypanosoma brucei* kinetoplast DNA minicircles. Protist, 154, 265–279. 13677453 10.1078/143446103322166554

[21] Hunt M, Silva ND, Otto TD, Parkhill J, Keane JA, Harris SR. 2015. Circlator: automated circularization of genome assemblies using long sequencing reads. Genome Biology, 16, 294. 26714481 10.1186/s13059-015-0849-0 PMC4699355

[22] Jensen RE, Englund PT. 2012. Network news: the replication of kinetoplast DNA. Annual Review of Microbiology, 66, 473–491. 10.1146/annurev-micro-092611-150057 22994497

[23] Kaufer A, Stark D, Ellis J. 2019. Evolutionary insight into the Trypanosomatidae using alignment-free phylogenomics of the kinetoplast. Pathogens, 8, 157. 31540520 10.3390/pathogens8030157 PMC6789588

[24] Kidane GZ, Hughes D, Simpson L. 1984. Sequence heterogeneity and anomalous electrophoretic mobility of kinetoplast minicircle DNA from *Leishmania tarentolae*. Gene, 27, 265–277. 6329906 10.1016/0378-1119(84)90071-4

[25] Li S-J, Zhang X, Lukeš J, Li B-Q, Wang J-F, Qu L-H, Hide G, Lai D-H, Lun Z-R. 2020. Novel organization of mitochondrial minicircles and guide RNAs in the zoonotic pathogen *Trypanosoma lewisi*. Nucleic Acids Research, 48, 9747–9761. 32853372 10.1093/nar/gkaa700 PMC7515712

[26] Lukes J, Guilbride DL, Votýpka J, Zíková A, Benne R, Englund PT. 2002. Kinetoplast DNA network: evolution of an improbable structure. Eukaryotic Cell, 1, 495–502. 12455998 10.1128/EC.1.4.495-502.2002 PMC117999

[27] Lukes J, Hashimi H, Zíková A. 2005. Unexplained complexity of the mitochondrial genome and transcriptome in kinetoplastid flagellates. Current Genetics, 48, 277–299. 16215758 10.1007/s00294-005-0027-0

[28] Naiff RD, Barrett TV. 2013. *Trypanosoma* (*Megatrypanum*) *lainsoni* n. sp. from *Mesomys hispidus* (Rodentia: Echimyidae) in Brazil: trypomastigotes described from experimentally infected laboratory mice. Parasite, 20, 51. 24309069 10.1051/parasite/2013049 PMC3853975

[29] Nantes WAG, Santos FM, Macedo GC de, Barreto WTG, Gonçalves LR, Rodrigues MS, Chulli JVM, Rucco AC, Assis W de O, Porfírio GE de O, Oliveira CE de, Xavier SC das C, Herrera HM, Jansen AM. 2021. Trypanosomatid species in *Didelphis albiventris* from urban forest fragments. Parasitology Research, 120, 223–231. 33079269 10.1007/s00436-020-06921-y

[30] Oliveira MM de, Ferrando CPR, Gómez-Hernández C, Oliveira KR de, Araújo IAC, Ribeiro PVA, Mineo TWP, Leiner NO, Mineo JR, Silva SM da. 2023. Prevalence of *Trypanosoma lainsoni* and its effects of parasitism on the health of non-volant small mammals from the Brazilian Cerrado. Parasitology Research, 122, 1509–1518. 37129625 10.1007/s00436-023-07851-1

[31] Ortiz PA, Garcia HA, Lima L, Silva FM da, Campaner M, Pereira CL, Jittapalapong S, Neves L, Desquesnes M, Camargo EP, Teixeira MMG. 2018. Diagnosis and genetic analysis of the worldwide distributed *Rattus*-borne *Trypanosoma* (*Herpetosoma*) *lewisi* and its allied species in blood and fleas of rodents. Infection, Genetics and Evolution, 63, 380–390. 10.1016/j.meegid.2017.09.001 28882517

[32] Quinlan AR, Hall IM. 2010. BEDTools: a flexible suite of utilities for comparing genomic features. Bioinformatics, 26, 841–842. 20110278 10.1093/bioinformatics/btq033 PMC2832824

[33] Ray DS. 1989. Conserved sequence blocks in kinetoplast minicircles from diverse species of trypanosomes. Molecular and Cellular Biology, 9, 1365–1367. 2542768 10.1128/mcb.9.3.1365-1367.1989 PMC362734

[34] Rodrigues MS, Lima L, Xavier SC das C, Herrera HM, Rocha FL, Roque ALR, Teixeira MMG, Jansen AM. 2019. Uncovering *Trypanosoma* spp. diversity of wild mammals by the use of DNA from blood clots. International Journal for Parasitology. Parasites and Wildlife, 8, 171–181. 30847276 10.1016/j.ijppaw.2019.02.004 PMC6389730

[35] Rusman F, Aramayo V, Floridia-Yapur N, Díaz AG, Ponce T, Hodi S, Aguirre JJ, Greif G, Berná L, Robello C, Diosque P, Tomasini N. 2025. Comparative maxicircle analysis in *Trypanosoma* species from the LSRM clade highlights patterns in an underexplored lineage. PLoS One, 20, e0332749. 40982458 10.1371/journal.pone.0332749 PMC12453231

[36] Rusman F, Díaz AG, Ponce T, Floridia-Yapur N, Barnabé C, Diosque P, Tomasini N. 2023. Wide reference databases for typing *Trypanosoma cruzi* based on amplicon sequencing of the minicircle hypervariable region. PLoS Neglected Tropical Diseases, 17, e0011764. 37956210 10.1371/journal.pntd.0011764 PMC10681310

[37] Rusman F, Floridia-Yapur N, Tomasini N, Diosque P. 2021. Guide RNA repertoires in the main lineages of *Trypanosoma cruzi*: High diversity and variable redundancy among strains. Frontiers in Cellular and Infection Microbiology, 11, 663416. 34136416 10.3389/fcimb.2021.663416 PMC8202002

[38] Rusman F, Tomasini N, Yapur N-F, Puebla AF, Ragone PG, Diosque P. 2019. Elucidating diversity in the class composition of the minicircle hypervariable region of *Trypanosoma cruzi*: New perspectives on typing and kDNA inheritance. PLoS Neglected Tropical Diseases, 13, e0007536. 31247047 10.1371/journal.pntd.0007536 PMC6619836

[39] Santos FM, Sano NY, Liberal SC, Dario MA, Nantes WAG, Alves FM, Silva AR da, De Oliveira CE, Roque ALR, Herrera HM, Jansen AM. 2022. Kinetoplastid species maintained by a small mammal community in the pantanal biome. Pathogens, 11, 1205. 36297262 10.3390/pathogens11101205 PMC9612235

[40] Selvapandiyan A, Duncan R, Mendez J, Kumar R, Salotra P, Cardo LJ, Nakhasi HL. 2008. A *Leishmania* minicircle DNA footprint assay for sensitive detection and rapid speciation of clinical isolates. Transfusion, 48, 1787–1798. 18564397 10.1111/j.1537-2995.2008.01798.x

[41] Simpson L, Thiemann OH, Savill NJ, Alfonzo JD, Maslov DA. 2000. Evolution of RNA editing in trypanosome mitochondria. Proceedings of the National Academy of Sciences of the United States of America, 97, 6986–6993. 10860961 10.1073/pnas.97.13.6986 PMC34374

[42] Simpson L, Douglass SM, Lake JA, Pellegrini M, Li F. 2015. Comparison of the mitochondrial genomes and steady state transcriptomes of two strains of the trypanosomatid parasite, *Leishmania tarentolae*. PLoS Neglected Tropical Diseases, 9, e0003841. 26204118 10.1371/journal.pntd.0003841 PMC4512693

[43] Tamura K, Stecher G, Kumar S. 2021. MEGA11: Molecular Evolutionary Genetics Analysis version 11. Molecular Biology and Evolution, 38, 3022–3027. 33892491 10.1093/molbev/msab120 PMC8233496

[44] Telleria J, Lafay B, Virreira M, Barnabé C, Tibayrenc M, Svoboda M. 2006. *Trypanosoma cruzi*: sequence analysis of the variable region of kinetoplast minicircles. Experimental Parasitology, 114, 279–288. 16730709 10.1016/j.exppara.2006.04.005

[45] Thorvaldsdóttir H, Robinson JT, Mesirov JP. 2013. Integrative Genomics Viewer (IGV): high-performance genomics data visualization and exploration. Briefings in Bioinformatics, 14, 178–192. 22517427 10.1093/bib/bbs017 PMC3603213

[46] Untergasser A, Cutcutache I, Koressaar T, Ye J, Faircloth BC, Remm M, Rozen SG. 2012. Primer3 – new capabilities and interfaces. Nucleic Acids Research, 40, e115. 22730293 10.1093/nar/gks596 PMC3424584

[47] Vallejo GA, Macedo AM, Chiari E, Pena SD. 1994. Kinetoplast DNA from *Trypanosoma rangeli* contains two distinct classes of minicircles with different size and molecular organization. Molecular and Biochemical Parasitology, 67, 245–253. 7870129 10.1016/0166-6851(94)00137-5

[48] Van den Broeck F, Savill NJ, Imamura H, Sanders M, Maes I, Cooper S, Mateus D, Jara M, Adaui V, Arevalo J, Llanos-Cuentas A, Garcia L, Cupolillo E, Miles M, Berriman M, Schnaufer A, Cotton JA, Dujardin J-C. 2020. Ecological divergence and hybridization of Neotropical *Leishmania* parasites. Proceedings of the National Academy of Sciences of the United States of America, 117, 25159–25168. 32958676 10.1073/pnas.1920136117 PMC7547230

[49] Vasimuddin Md, Misra S, Li H, Aluru S. 2019. Efficient architecture-aware acceleration of BWA-MEM for multicore systems. 2019 IEEE International Parallel and Distributed Processing Symposium (IPDPS). pp. 314–324.

[50] Veas F, Cuny G, Brenière SF, Tibayrenc M. 1990. Subspecific kDNA probes for major clones of *Trypanosoma cruzi*. Acta Tropica, 48, 79–82. 1980806 10.1016/0001-706x(90)90067-a

[51] Wick RR, Holt KE. 2022. Polypolish: Short-read polishing of long-read bacterial genome assemblies. PLoS Computational Biology, 18, e1009802. 35073327 10.1371/journal.pcbi.1009802 PMC8812927

[52] Ye J, Coulouris G, Zaretskaya I, Cutcutache I, Rozen S, Madden TL. 2012. Primer-BLAST: a tool to design target-specific primers for polymerase chain reaction. BMC Bioinformatics, 13, 134. 22708584 10.1186/1471-2105-13-134 PMC3412702

