# Supplementary material for: Deciphering Trypanosoma lainsoni kDNA minicircles: insights into genetic diversity, mRNA editing, and molecular diagnosis
Source: Parasite. 2026 Jun 3;33:34. doi: 10.1051/parasite/2026034 (PMC13233029; doi:10.1051/parasite/2026034)
Supplement: Supplementary file 1 — Editing cascades of ATPase 6, CR3, CR4, ND3, ND7, ND8, ND9, and RPS12 mRNAs, predicted from the sequencing reads of each T. lainsoni isolate: Le29, Ca47, and Ca37. Guide RNAs are shown aligned below the mRNA sequences according to their editing position. The x-axis represents the mRNA position, while the y-axis shows the cumulative number of gRNA classes up to that position. gRNA classes are color-coded according to sequence abundance as follows: red (1–19 reads), black to green (20–1,000 reads), and light green (>1,000 reads). [file parasite-33-34-s1.pdf]

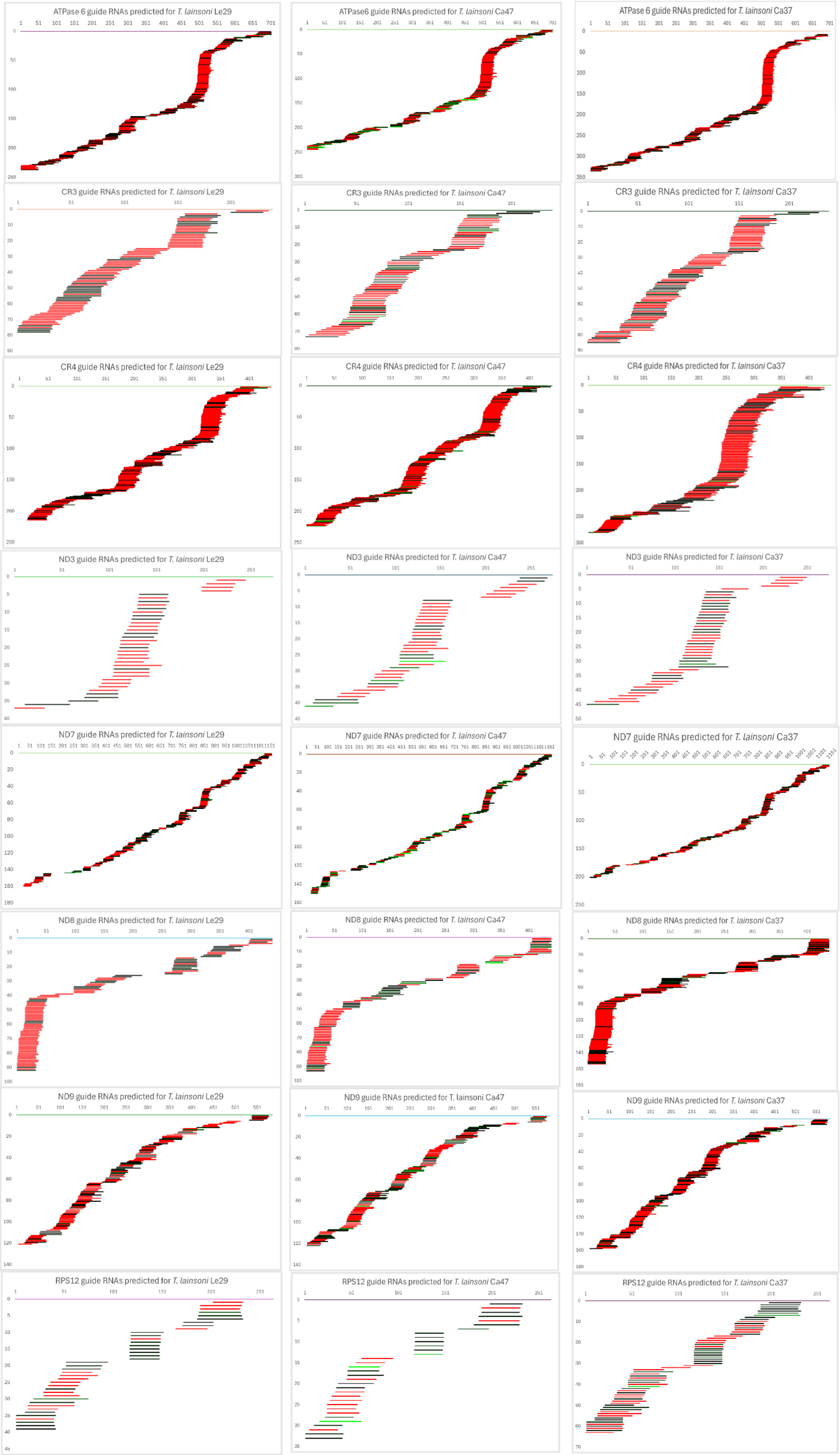

Editing cascades predicted for *ATP synthase subunit 6* (*ATPase 6)*, unknown-function *CR3* (*CR3*), unknown-function *CR4* (*CR4*), *NADH dehydrogenase subunit 3* (*ND3*), *NADH dehydrogenase subunit 7* (*ND7*), *NADH dehydrogenase subunit 8* (*ND8*), *NADH dehydrogenase subunit 9* (*ND9*), and *ribosomal protein S12* (*RPS12*) genes from the sequencing reads of each *T. lainsoni* isolate.
