# Supplementary material for: Deciphering Trypanosoma lainsoni kDNA minicircles: insights into genetic diversity, mRNA editing, and molecular diagnosis
Source: Parasite. 2026 Jun 3;33:34. doi: 10.1051/parasite/2026034 (PMC13233029; doi:10.1051/parasite/2026034)
Supplement: Supplementary file 2 — Supplementary Table 1: Summary of contigs obtained by KOMICS pipeline. [file parasite-33-34-s2.pdf]

Supplementary Table 1. Summary of contigs obtained by KOMICS pipeline.

Contigs		Assembled	Corrected	Mean Length	Median Length
Le29	Total	872	838	1,309.0	738.5
	Circularized	225	220	1,073.1	1,179.0
Ca37	Total	1,278	1,174	1,122.6	729.0
	Circularized	259	253	1,004.8	1,177.0
Ca47	Total	661	642	1,465.1	741.5
	Circularized	166	165	1,150.1	1,184.0
T. lainsoni	Total	2,811	2,654	1,264.3	735.5
	Circularized	650	638	1,065.9	1,180.0
