# Supplementary material for: Deciphering Trypanosoma lainsoni kDNA minicircles: insights into genetic diversity, mRNA editing, and molecular diagnosis
Source: Parasite. 2026 Jun 3;33:34. doi: 10.1051/parasite/2026034 (PMC13233029; doi:10.1051/parasite/2026034)
Supplement: Supplementary file 3 — Supplementary Table 2: Mapping statistics of Illumina reads against contigs obtained by KOMICS. [file parasite-33-34-s3.pdf]

Supplementary Table 2. Mapping statistics of Illumina reads against contigs obtained by KOMICS.

Statistics	Le29	Ca37	Ca47
Reads	80,382,286	68,596,926	85,491,750
Mapped reads	6,698,409	4,995,112	7,752,507
Mapped reads >Q20	3,846,751	3,139,677	5,063,920
Reads (CSB-3)	135,593	223,658	422,324
Mapped reads (CSB-3)	133,694	220,637	418,723
Mapped reads >Q20 (CSB-3)	120,181	192,190	378,889
Mean Depth	151.75	173.45	513.47
Minimum Depth	14.20	9.51	25.59
Maximum Depth	1226.93	741.94	1947.20
Mapping percentage	8.33%	7.28%	9.07%
Mapping percentage >Q20	4.79%	4.58%	5.92%
Mapping percentage (CSB-3)	98.60%	98.65%	99.15%
Mapping percentage >Q20 (CSB-3)	88.63%	85.93%	89.72%
