# Supplementary material for: Deciphering Trypanosoma lainsoni kDNA minicircles: insights into genetic diversity, mRNA editing, and molecular diagnosis
Source: Parasite. 2026 Jun 3;33:34. doi: 10.1051/parasite/2026034 (PMC13233029; doi:10.1051/parasite/2026034)
Supplement: Supplementary file 4 — Supplementary Table 3: Summary of size filtered T. lainsoni minicircle contigs. [file parasite-33-34-s4.pdf]

Supplementary Table 3. Summary of size filtered *T. lainsoni* minicircle contigs.

	Contigs	Short contigs	Mean	Median	Std(σ)	Long contigs	Mean	Median	Std(σ)
Le29	214	50	592.4	593	4.7	164	1,183.5	1,183	10.0
Ca37	249	84	593.8	594	4.9	165	1184.3	1,183	9.2
Ca47	158	21	596.0	596	5.0	137	1,185.6	1,186	9.2
T. lainsoni	621	155	593.6	594	4.9	466	1,184.4	1,184	9.5

Contigs were filtered by size (<1,600 bp) and grouped in two categories according to their mean length
